# Galectin-9 Induced by Dietary Prebiotics Regulates Immunomodulation to Reduce Atopic Dermatitis Symptoms in 1-Chloro- 2,4-Dinitrobenzene (DNCB)-Treated NC/Nga Mice

**DOI:** 10.4014/jmb.2005.05017

**Published:** 2020-07-23

**Authors:** Jeong A Kim, Sung Hak Kim, In Sung Kim, Da Yoon Yu, Gwang Il Kim, Yang Soo Moon, Sung Chan Kim, Seung Ho Lee, Sang Suk Lee, Cheol-Heui Yun, In Soon Choi, Kwang Keun Cho

**Affiliations:** 1 Department of Animal Resources Technology, Gyeongnam National University of Science and Technology, Jinju 52725, Republic of Korea; 2 Department of Animal Science, Chonnam National University, Gwangju 61186, Republic of Korea; 3 Department of Animal Science & Biotechnology, Gyeongnam National University of Science and Technology, Jinju 52725, Republic of Korea; 4 Department of Biochemistry, Institute of Cell Differentiation and Aging, College of Medicine, Hallym University, Chuncheon 24252, Republic of Korea; 5 Department of Nano-Bioengineering, Incheon National University, Incheon 22012, Republic of Korea; 6 Department of Animal Science and Technology, Sunchon National University, Sunchon 57922, Republic of Korea; 7 Department of Agricultural Biotechnology and Research Institute of Agriculture and Life Sciences, Seoul National University, Seoul 08826, Republic of Korea; 8 Department of Life Science, Silla University, Busan 46958, Republic of Korea

**Keywords:** Gut microbiota, immunomodulation, prebiotics, Th1 cells, Th2 cells, Treg cells

## Abstract

Atopic dermatitis (AD) is a skin disorder that causes chronic itch. We investigated the inhibitory effects of a mixture of prebiotic short-chain galacto-oligosaccharides and long-chain fructo- oligosaccharides (scGOS/lcFOS), inulin, or β-glucan on AD development in 1-chloro-2,4- dinitrobenzene (DNCB)-treated NC/Nga mice. Mice were randomly assigned to six groups: untreated mice, AD control, positive control (DNCB-treated NC/Nga mice fed a dietary supplement of Zyrtec), and DNCB-treated NC/Nga mice fed a dietary supplement of prebiotics such as scGOS/lcFOS (T1), inulin (T2), or β-glucan (T3). The prebiotic treatment groups (T1, T2, and T3) showed suppression of AD symptoms, Th2 cell differentiation, and AD-like skin lesions induced by DNCB. In addition, prebiotic treatment also reduced the number of microorganisms such as *Firmicutes*, which is associated with AD symptoms, and increased the levels of *Bacteroidetes* and *Ruminococcaceae*, which are associated with alleviation of AD symptoms. Our findings demonstrate the inhibitory effects of prebiotics on AD development by improving the Th1/Th2 cytokine balance and beneficial symbiotic microorganisms in in vitro and in vivo models.

## Introduction

Atopic dermatitis (AD) is a chronic and inflammatory autoimmune disorder that results in severe itch and eczematous skin lesions [1]. The development of autoimmune disorders is associated with a defective immune system, loss-of-function variants of the epidermal barrier protein filaggrin [2], and disruption of the gut microbial community. In AD, the immunological imbalance between type 1 and type 2 helper T cells (Th1 and Th2 cells, respectively) results in an increase in antigen-specific IgE levels mediated via cytokines such as IL-4, IL-5, and IL- 14 [3-5].

Recent findings suggested that intestinal resident microorganisms play an important role in regulating the gut mucosal immune system and protection against pathogen invasion [6]. Microbial dysbiosis due to diet or antibiotic treatment, infection, and metabolic alteration results in serious health problems including intestinal inflammation [7]. In particular, loss of microbial diversity has a substantial impact on chronic inflammatory skin diseases [4].

Prebiotics are food ingredients selectively metabolized by gut bacteria, and they stimulate the growth of beneficial microorganisms [8], resulting in changes in the composition of intestinal microorganisms [9]. Fructo- oligosaccharides (FOS), inulin, beta-glucan, and galacto-oligosaccharides (GOS) represent sources of prebiotic dietary fiber [10]. Exposure to a mixture of short chain GOS (scGOS) and ling chain FOS (lcFOS) reduces the incidence of AD. In addition, prebiotics improve the symptoms of AD and facilitate the induction of innate responses such as toll-like receptor agonists and cytokines [11, 12]. However, the effects of prebiotic dietary fiber on AD-like skin lesions in mice and alterations in gut microbiota have yet to be investigated.

Thus, in the present study, we investigated whether prebiotics can reduce the symptoms of 1-chloro-2,4- Dinitrobenzene (DNCB)-induced AD-like skin lesions in NC/Nga mice by changing the gut microbiota and elucidated the underlying immune mechanism.

## Materials and Methods

### Animals

Five-week-old male NC/Nga mice (Central Lab, Animal Inc., Republic of Korea) were maintained at room temperature (22 ± 1°C), with a 12-h light–dark cycle during the experimental period and were provided ad libitum access to food (AIN-76A; Central Lab) and water. Mice were fed with scGOS (Neo Cremar Co., Republic of Korea)/lcFOS (Cell Biotech Co., Republic of Korea) at a ratio of 9:1 to 1% of the diet, and inulin (Vixxol Co., Republic of Korea) and β-glucan (Glucan Co., Republic of Korea) were supplemented at a rate of 1% of the diet. Zyrtec (0.2 mg/kg) (KyungDong Pharm Co., Republic of Korea) was provided in the daily diet as a positive control to alleviate symptoms of atopic dermatitis. After a one-week preliminary experimental period and a four-week AD induction period, the experimental animals were randomly divided into six groups with 6 animals per group: (1) Untreated control group (C: basal diet), (2) AD control group (N: basal diet + DNCB-induced group), (3) Positive control group (P: basal diet + Zyrtec + DNCB-induced group), (4) scGOS/lcFOS group (T1: basal diet + scGOS/lcFOS + DNCB-induced group), (5) inulin group (T2: basal diet + inulin + DNCB-induced group), and (6) β-glucan group (T3: basal diet + β-glucan + DNCB-induced group). The experimental protocol was approved by the Institutional Animal Care Board of Gyeongnam National University of Science and Technology (Approval No. 2017-8).

### AD Model

**Fig. 1:**
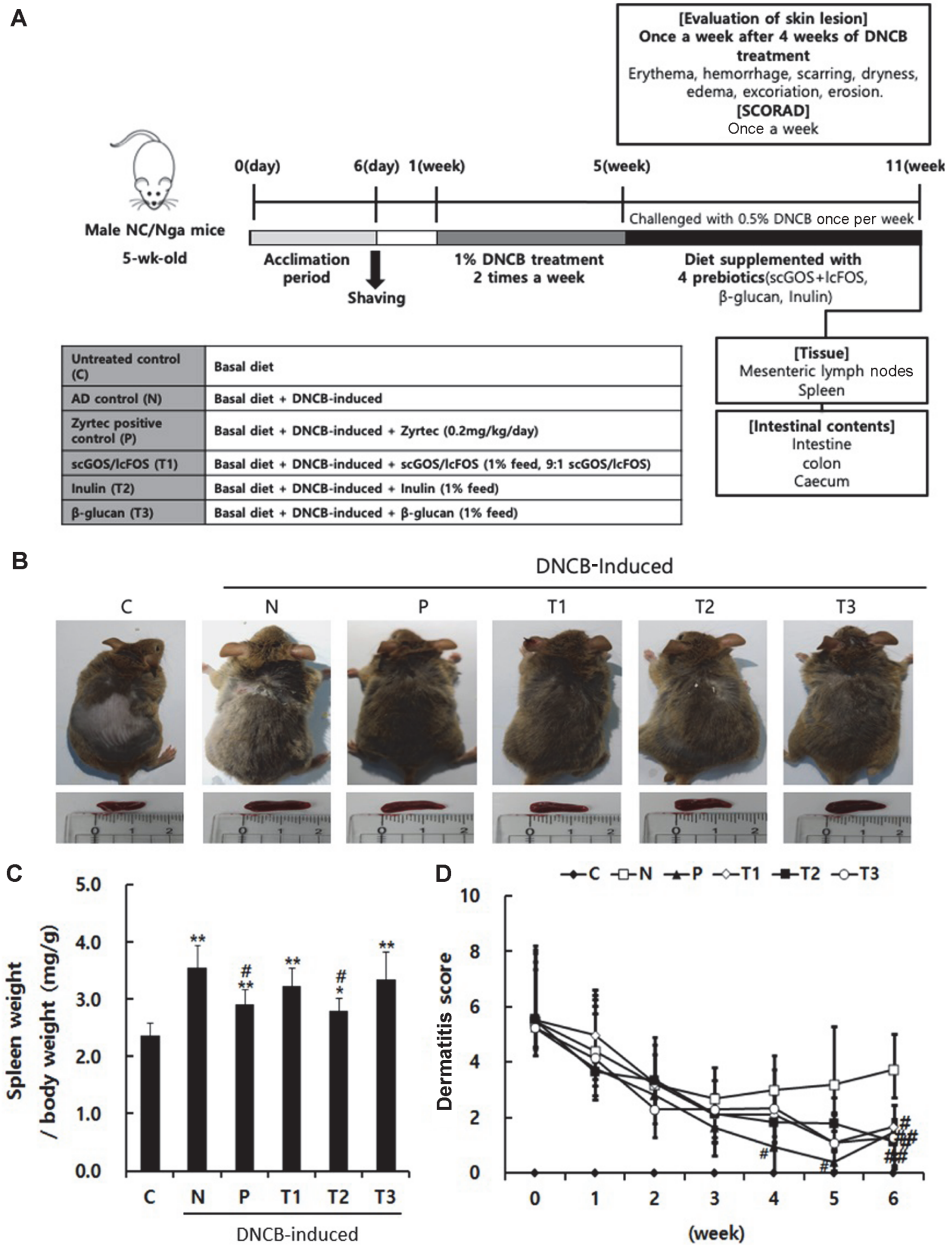
Effect of prebiotics on DNCB-induced AD-like symptoms in NC/Nga mice. (**A**) Experimental design (**B**) DNCB-induced AD-like skin lesions and spleen size. (**C**) Spleen size-to-body weight ratio was evaluated. (**D**) The dermatitis score of AD-like skin lesions was measured to evaluate itch, erythema/hemorrhage, scaling/dryness, edema, and excoriation/ erosion. C: Control, N: Negative control (DNCB-induced), P: Zyrtec positive control (DNCB-induced + Zyrtec), T1: scGOS/ lcFOS (DNCB-induced + scGOS/lcFOS), T2: Inulin (DNCB-induced + Inulin), T3: β-glucan (DNCB-induced + β-glucan); *p < 0.05 versus control group; **p < 0.01 versus control group; #*p* < 0.05 versus negative control group; ##*p* < 0.01 versus negative control group. Data represent means ± SD of 5 replicates.

According to the method described by Shin *et al*. [13], after a one-week preliminary experimental period, AD- like skin lesions were induced in NC/Nga mice by using 1-chloro-2,4-dinitrobenzene (DNCB; Sigma, USA). The dorsal dermis was shaved with an electronic clipper one day before treatment with DNCB. DNCB solution was prepared at a concentration of 1% in an acetone:olive oil suspension (3:1), and used to treat the dorsal skin of mice twice a week for 4 weeks. After 4 weeks of AD induction, the experimental animals were randomly divided into 6 groups and fed prebiotics for 6 weeks. During the feeding, mice were challenged with 0.5% DNCB once a week (Fig. 1A).

### Evaluation of AD

The AD severity score was assessed visually once a week after 4 weeks of DNCB treatment. Scores of 0 (none), 1 (mild), 2 (moderate), and 3 (severe) were measured for each of the four symptoms: erythema/hemorrhage, scarring/dryness, edema, and excoriation/erosion. The sum of the individual scores indicating clinical severity represented the dermatitis score.

### Separation and Analysis of RNA from Mouse Mesenteric Lymph Nodes (MLN)

To evaluate the immunomodulatory effects of prebiotics, atopic-induced NC/Nga mice were sacriﬁced. The mesenteric lymph node (MLN) and spleen were separated. TRIzol was added to the MLN of NC/Nga mice and homogenized using SilentCrusher M (Heidolph, Germany). According to the method presented by Chomczynski and Sacchi [14], RNA was extracted and stored at -20°C until cDNA synthesis. The cDNA was synthesized using reverse transcription-polymerase chain reaction (RT-PCR) kits (TaKaRa Co., Japan) according to the manufacturer’s instructions. The primer sequences are listed in Table 1 [15-19]. Glyceraldehyde-3-phosphate dehydrogenase (GAPDH) was used as the reference gene.

### Western Blot Analysis

Whole cell extracts were prepared with RIPA lysis buffer (150 mM NaCl, 1% NP-40, 0.1% SDS, 1% sodium deoxycholate, 50 mM Tris [pH 8.0]) containing 1 mM NaF, 1 mM Na_3_VO_4_, and protease inhibitors. Proteins were quantified using the Bradford assay reagent (Bio-Rad) according to the manufacturer’s instructions. Proteins (20– 40 μg) were separated by 10% sodium dodecyl sulfate-polyacrylamide gel electrophoresis and transferred to polyvinylidene fluoride membranes (EMD Millipore, USA). Membranes were blocked with 5% nonfat milk and incubated with the following antibodies: Galectin-9 (ab194338; 1:1000 dilution; Abcam), TLR 9 (ab134368, 1:1000dilution; Abcam), and GAPDH (#2118, 1:10,000; Cell Signaling). Membranes were then incubated with horseradish peroxidase-conjugated secondary antibodies and visualized with Immobilon Western Chemiluminescent HRP Substrate (EMD Millipore).

### Cell Lines and Culture Conditions

The human colorectal adenocarcinoma cell line HT29 was purchased from the American Type Culture Collection (ATCC) and maintained in RPMI1640 with stable glutamine (Biowest) enriched with 10% fetal bovine serum (FBS)(Biowest), 1% penicillin/streptomycin (Welgene), and 25 mM HEPES (Shimakyu Co., Ltd., Japan), 25 mM NaHCO_3_ (Shimakyu Co., Ltd.,) at 37°C with 5% CO_2_. Isolated MNL lymphocytes were cultured in RPMI 1640 with L-glutamine and sodium bicarbonate (Sigma), 10% FBS (Biowest), 1% penicillin/streptomycin (Welgene), and 0.05 mM 2-β-mercaptoethanol (Sigma) at 37°C in the presence of 5% CO_2_. For western blot analysis, Gos/Fos, inulin, and β-glucan were treated in the presence of 0, 0.25, 0.5, 1, 2, 5 % w/v, respectively for 12 h.

### Analysis of Gut Microbiota

On the last day of the experiment, the NC/Nga mice were sacriﬁced and fecal samples were collected from them and stored at -80°C until being used for genomic DNA (gDNA) extraction. DNA was extracted using fecal DNA MiniPrep kits (Zymo Research, USA), and the sequences were analyzed using Illumina MiSeq (ChunLab, Inc., Republic of Korea).

### Processing of Sequencing Data

The pyrosequencing data for the 16S rRNA gene sequences were processed as we described previously (Kim *et al*., 2018). To filter low quality sequences, reads < 300bp were removed, and the trimmed sequencing reads were clustered at 97% sequence similarity level using a TBC clustering algorithm to pick operational taxonomic units (OTUs; Lee *et al*., 2012). In order to identify taxonomy, representative sequences were selected from the OTUs, which are assigned to taxonomic positions based on the highest pairwise similarity among the top five BLASTN hits in the EzTaxon-e database (Kim *et al*., 2012). Myers-Miller global pairwise alignment was used to calculate the nucleotide sequence similarity between the query and candidate species (Myers & Miller, 1988). The TBC clustering algorithm was used to analyze the cladogram (Lee *et al*. 2012). The read numbers in each sample were normalized by random subsampling. The analysis of phylogenetic differences was performed using the CL community program provided by ChunLab. Statistical significance in relative abundance among different groups was calculated through variance analysis using the General Linear Model procedure of the SAS (Ver. 9.1) and means comparison using Duncan’s multiple range tests at 5% significance level.

### Quantitative Real-Time PCR

As mentioned above, DNA was extracted from NC/Nga mice fecal samples. Quantitative PCR (qPCR) was performed using Rotor-Gene SYBR Green PCR Kits (QIAGEN GmbH, Germany). The primer sequences of the microorganisms used for qPCR analysis are listed in Table 2 [20-23]. Universal Reference Gene (URG) method was used.

### Statistical Analysis

The variance of the results obtained via repeated experiments was analyzed using SPSS 12.0 (SPSS, Inc., USA). The signiﬁcance of the results was tested using Duncan’s multiple range test at *p* < 0.05, and intergroup signiﬁcance was tested using the independent-sample t-test at *p* < 0.05 and *p* < 0.01. All results are indicated as mean ± standard deviation.

## Results

### Allergic Symptoms in Mice

To investigate the effects of prebiotics on DNCB-induced AD symptoms in mice, the mice were treated with scGOS/lcFOS (T1), inulin (T2), or β-glucan (T3) once a week for six weeks (Fig. 1A). Body weight and body weight gain were decreased in the DNCB-induced AD control (N), scGOS/lcFOS (T1), and inulin (T2) groups compared with the untreated control (C) group. However, positive control (P) and β-glucan (T3) groups showed no significant decrease in body weight and body weight gain (Supplemental File). Furthermore, average daily dietary feed intakes (ADFI) were not different between the treatments (Supplemental File). Feed efficiency decreased with all treatments except in the β-glucan groups (T3) compared with untreated control (C) group (Supplemental File).

DNCB-induced AD control mice (N) showed red spots, edema, and bleeding. Prebiotic (T1-T3) and Zyrtec (P) groups showed a clear decrease in these symptoms (Fig. 1B). Because AD influences the weight of immune organs such as the spleen by modulating various immune responses [24], we next investigated the effect of these prebiotics on spleen weight in the AD mouse model. A decrease in spleen weight was observed in the Zyrtec (P) and inulin (T2) groups compared with the DNCB-induced AD control (N) group (Fig. 1C). Skin lesions were determined according to the SCORAD index to confirm the effect of prebiotics on AD. The index was not clearly different between the DNCB-induced AD control (N) and prebiotic (T1–T3) groups at 3 weeks. However, positive control (P) and prebiotic (T1~T3) groups showed a gradual decrease in the index at 6 weeks compared with that of the DNCB-induced AD control (N) group (Fig. 1D).

### T Cell Immune Responses and Skin Barrier-Related Gene Expression

**Fig. 2:**
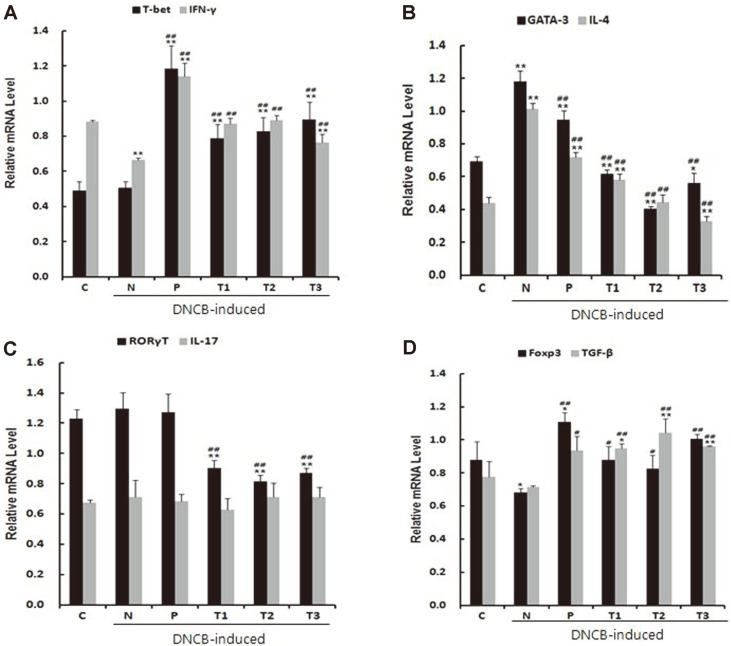
Effect of prebiotics on the expression of transcription factors and cytokines. T-cell polarization in mesenteric lymph nodes (MLN) was evaluated by analyzing the expression of T-bet, IFN-γ (Th1, A), GATA-3, IL-4 (Th2, B), RORγT, IL-17 (Th17, C) and Foxp3, TGF-β (Treg, D). C: untreated control, N: AD control (DNCB-induced), P: Zyrtec-positive control (DNCB-induced + Zyrtec), T1: scGOS/lcFOS (DNCB-induced + scGOS/lcFOS), T2: Inulin (DNCB-induced + Inulin), T3: β-glucan (DNCB-induced + β-glucan); *p < 0.05 versus control group, **p < 0.01 versus control group, #*p* < 0.05 versus negative control group, ##*p* < 0.01 versus negative control group. Data represent means ± SD of 4 replicates.

In order to examine the effects of prebiotics on the T cell populations including Th1, Th2, Th17, and Treg, we analyzed the mRNA expression of their specific transcription factors and cytokines such as T-bet, GATA3, RORγt, Foxp3, interferon (IFN)-γ, interleukin(IL)-4, IL-17, and TGF-β, respectively, in the mesenteric lymph nodes (MLN) derived from the DNCB-induced AD mouse model. The AD control (N) group showed a significant decrease in the mRNA expression of IFN-γ and Foxp3, which are transcription factors of Th1 and Treg, respectively, compared with the untreated control (C) group. The prebiotic (T1~T3) and Zyrtec (P) groups clearly showed an increase in mRNA expression of Th1 and Treg-specific transcription factors and cytokines such as T- bet, IFN-γ, Foxp3, and TGF-β compared with the DNCB-induced AD control (N) group (Figs. 2A and 2D). However, treatments with prebiotics (T1~T3) induced a significant decrease in the mRNA expression of Th2- specific transcription factor GATA3 and cytokine IL-4, which were increased in the DNCB-induced AD control (N) (Fig 2B). RORγt, a Th17 transcription factor associated with acute AD [25], was markedly reduced in prebiotic (T1~T3) groups (Fig. 2C). However, no change in IL-17 expression was detected between the different treatments.

The development of allergic symptoms is regulated by a balance between Th1/Treg and Th2 cell populations. It is known that galectin-9 alleviates the allergic symptoms by promoting Th1/Treg cell differentiation, while thymic stromal lymphopoietin (TSLP) aggravates the symptoms via differentiation of naïve cells into Th2 cells [13].

**Fig. 3:**
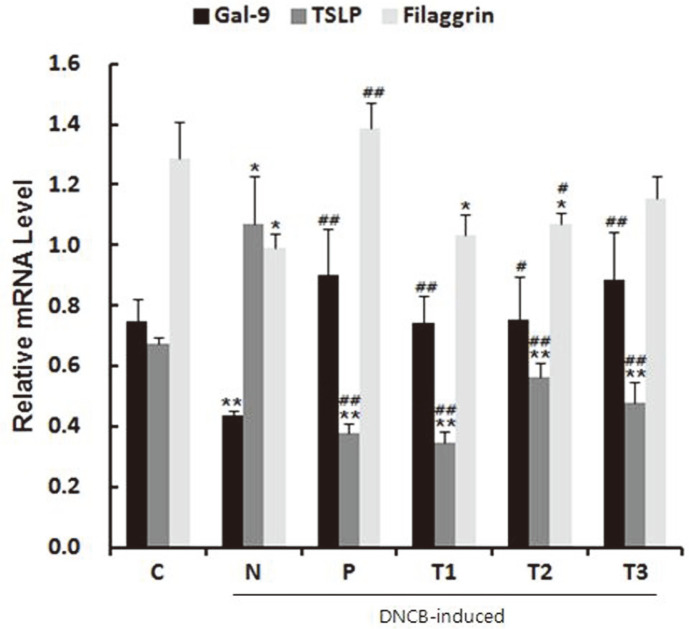
Effect of prebiotics on the expression of galectin-9, filaggrin, and TSLP in the mesenteric lymph nodes (MLN). C: Untreated control, N: AD control (DNCB-induced), P: Zyrtec-positive control (DNCB-induced + Zyrtec), T1: scGOS/lcFOS (DNCB-induced + scGOS/lcFOS), T2: Inulin (DNCB-induced + Inulin), T3: β-glucan (DNCB-induced + β- glucan); *p < 0.05 versus control group, **p < 0.01 versus control group, #*p* < 0.05 versus negative control group, ##*p* < 0.01 versus negative control group. Data represent means ± SD of 4 replicates.

The DNCB-induced AD control (N) group showed a marked decrease in the mRNA expression of galectin-9 compared with the untreated control (C) group, which was significantly increased after prebiotic (T1–T3) treatment. However, the level of TSLP mRNA was dramatically increased in the AD (N) group compared with the untreated control (C) group and decreased in the prebiotic (T1–T3) groups. We next analyzed the expression of the filaggrin gene, which is associated with skin barrier function and decreased AD symptoms [26]. The mRNA level of filaggrin was significantly decreased in the DNCB-induced AD control (N) compared with the untreated control (C) group, and was increased in the inulin (T2) group. These results indicate that prebiotics regulate the immune function by balancing the levels of Th1, Th2, and Treg cells, and decrease Galactin-9, which increases Th1 and Treg cell differentiation, and TSLP, which increases Th2 cell differentiation, indicating amelioration of AD symptoms (Fig. 3).

### TLR-9 and Galactin-9 Protein Expression Levels in HT29 Cell Lines

**Fig. 4:**
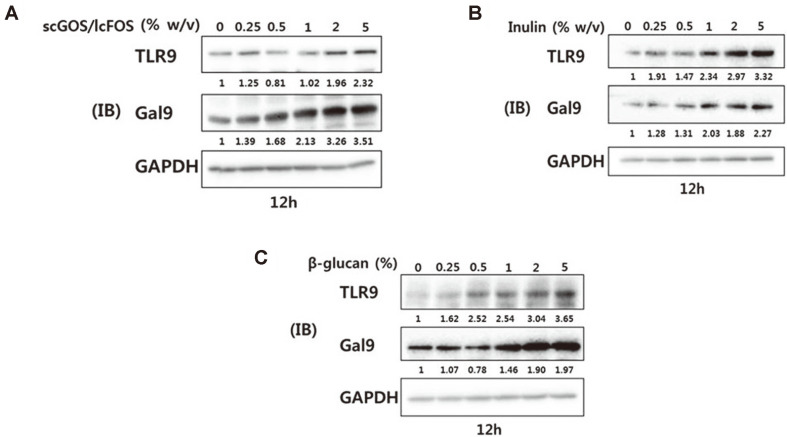
Prebiotics increase TLR-9 and Gal-9 protein expression in HT29 cell lines (A, B, C). Western blot analysis showed a dose-dependent increase in TLR-9 and Gal-9 protein levels in HT29 cells following treatment with scGOS/lcFOS (9:1 w/v mixture), inulin, and β-glucan for 12 h, respectively.

TLR9 enhances Th1 and Treg cell populations via IFN-γ, resulting in the prevention and treatment of allergic diseases [27]. Thus, we analyzed the protein expression of TLR-9 and galectin-9 after treatment with prebiotics in intestinal epithelial cells (HT-29 cells) (Fig. 4). Prebiotic treatments resulted in increased protein expression of TLR-9 and galectin-9 in HT-29 cells in a concentration-dependent manner. Thus, scGOS/lcFOS, inulin, and β- glucan are immunomodulated via activation of galectin-9 and TLR-9.

### Diversity Analysis of Gut Microbiota

**Fig. 5:**
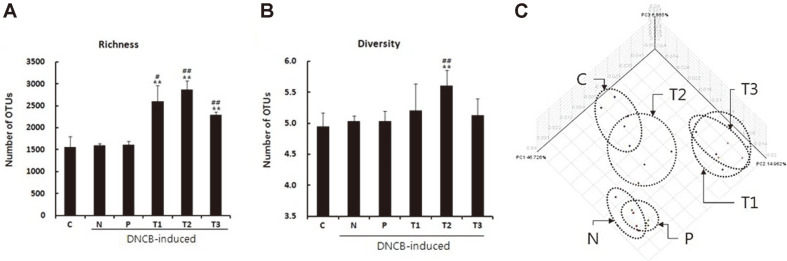
Intestinal microbiota richness and diversity was increased with prebiotic treatment. (**A**) Richness, (**B**) Diversity, and (C) Principal coordinate analysis (PcoA) of community structures using a Fast UniFrac distance matrix. C: Untreated control, N: AD control (DNCB-induced), P: Zyrtec-positive control (DNCB-induced + Zyrtec), T1: scGOS/lcFOS (DNCB-induced + scGOS/lcFOS), T2: Inulin (DNCB-induced + Inulin), T3: β-glucan (DNCB-induced + β-glucan). Percentage of variation explained by principal component 1 (PC1): 46.726%; percentage of variation explained by principal component 2 (PC2): 14.962%; percentage of variation explained by principal component 3 (PC3): 6.865%. *p < 0.05 versus control group, **p < 0.01 versus control group, #*p* < 0.05 versus negative control group, ##*p* < 0.01 versus negative control group. Data represent means ± SD of 4 replicates.

In the early stages of life, dysbiosis in the intestinal microbiome is the main cause of allergic diseases [28, 29]. Therefore, we analyzed the diversity of fecal microbiota in mice via 16S rRNA gene sequencing (Table 3 and Figs. 5A and 5B). The prebiotics (T1-T3) increased the intestinal microbial diversity compared with AD (N) and Zyrtec (P) groups (Table 3). In addition, intestinal microbial diversity was significantly increased in the scGOS/ lcFOS (T1) and inulin (T2) groups at the species level compared with the untreated control (C) group (Table 3). The Chao1 index indicated that the richness of intestinal microbiota in the group treated with prebiotics (T1-T3) was significantly increased compared with negative (N) and positive control (P) groups (Fig. 5A). The Shannon index showed that the inulin (T2) group had higher microbial diversity than the other groups (Fig. 5B).

### Principal Coordinate Analysis (PCoA) Analysis of Gut Microbiota

The overall structural changes of the intestinal microbiota were then analyzed using principal coordinate analysis (PCoA). The PcoA plots showed that microbial communities from untreated control (C) and inulin (T2) groups, scGOS/lcFOS (T1) and β-glucan (T3) groups, AD control (N) and Zyrtec (P) groups were similar to each other (Fig. 5C), which indicates that prebiotics alleviate AD via changes in the intestinal microbial community. The Zyrtec (P) group showed a microbial community structure similar to that of AD control (N) group, suggesting that Zyrtec has no attenuating effect on AD symptoms due to intestinal microbial changes. These results suggest that changes in intestinal microbial communities may affect the induction and mitigation of AD.

### Analysis of Intestinal Microbial Changes in Mice

Intestinal microorganisms constitute the immune system, and affect the incidence of various diseases, including AD [30]. Therefore, the effects of prebiotics on changes in gut microbiota were analyzed (Table 4). In general, an increase in *Firmicutes* and a decrease in *Bacteroidetes* have been observed in children with eczema or AD [30, 31].The proportion of *Firmicutes* was increased in DNCB-induced AD control (N) group compared with the untreated control (C) group, whereas that of *Bacteroidetes* was decreased. Prebiotics (T1~T3) caused a significant decrease of *Firmicutes*, but induced an increase in *Bacteroides*. Furthermore, treatment with scGOS/lcFOS (T1) and β-glucan (T3) decreased the high levels of *Clostridia* [32] in patients with AD, compared with the negative group (N). Analysis of intestinal microorganisms at the family level revealed that *Lachnospiraceae* and *Ruminococcaceae* accounted for 8.93–70.47% of intestinal microorganisms. The incidence of *Lachnospiraceae* is increasd in allergy patients [33], and that of *Ruminococcaceae* is decreased in eczema cases [34]. *Ruminococcaceae* were significantly increased in groups treated with prebiotics (T1-T3) compared with AD control (N) group, which was consistent with previous findings suggesting that prebiotic treatment controlled AD symptoms.

### Immunomodulatory Effects by Intestinal Microbial

Groups of intestinal microorganisms such as *Clostridia cluster IV* and *XIVa* exhibit immunomodulatory effects via induction of Treg cells [35]. The immunomodulatory effects of *C. cluster IV* and *XIVa* on intestinal microorganisms were analyzed (Table 5). *Ruminococcus* species belonging to *Clostridia cluster XIVa* was significantly increased in the prebiotic (T1~T3) group compared with AD control (N) group at the genus level. In addition, *R. lactaris* was significantly increased in the β-glucan (T3) group compared with the AD control (N) group at the species level. *Coprococcus comes* was significantly higher in the scGOS/lcFOS (T1) group than in the AD control (N) group. Thus, prebiotics induce Treg cells via *Clostridia IV* and *XIVa* clusters, resulting in immunomodulatory effects.

### Functional Bacteria Analysis by qPCR

**Fig. 6:**
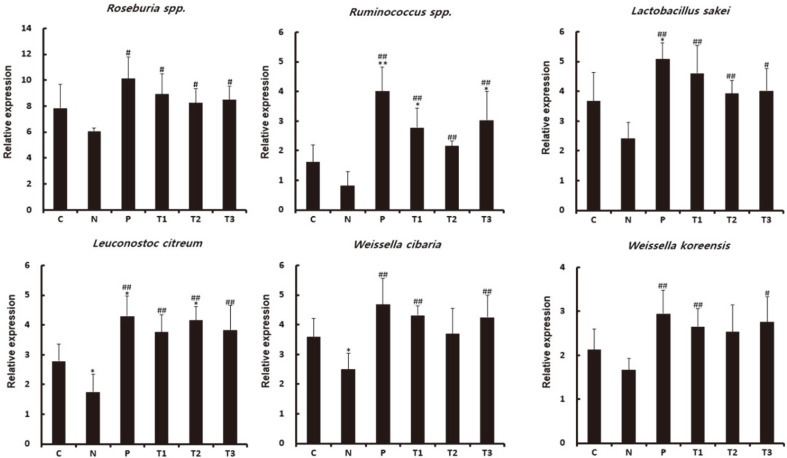
Measurement of the abundance of functional bacteria in the feces of prebiotic-treated groups by quantitative PCR. (**A**, **B**) Butyrate-producing bacteria; (**C**-**F**) lactic-acid bacteria; C: Untreated control, N: AD control (DNCB-induced), P: Zyrtec-positive control (DNCB-induced + Zyrtec), T1: scGOS/lcFOS (DNCB-induced + scGOS/lcFOS), T2: Inulin (DNCB-induced + Inulin), T3: β-glucan (DNCB-induced + β-glucan); *p < 0.05 versus control group, **p < 0.01 versus control group, #*p* < 0.05 versus negative control group, ##*p* < 0.01 versus negative control group. Data represent means ± SD of 4 replicates.

Next, we used qPCR to investigate the changes in intestinal populations of functional microorganisms (Fig. 6). We found that the levels of butyrate-producing microorganisms *Roseburia* spp*.* and *Ruminococcus* spp*.* were significantly increased in prebiotic (T1–T3) groups compared with the atopy-inducer (N) group, and *Lactobacillus sakei, Leuconostoc citreum, Weissella cibaria,* and *W. koreensis* were significantly increased in the prebiotic (T1– T3) group.

## Discussion

In this study, we investigated the effects of prebiotics on the development of AD. In order to establish an AD mouse model, we treated the dorsal skin of NC/Nga mice with DNCB and detected the generation of AD-like skin lesions, such as red spots, edema, and bleeding. We found that prebiotic supplementation resulted in suppression of AD symptoms via reduction in spleen weight and SCORAD index. Increased spleen weight is associated with AD symptoms [36]. Recent studies suggested that AD is triggered by an imbalance between Th1, Th2, and Treg cell-related immune responses [37]. Abnormal expression of IFN-γ, IL-4, and IL-5 by T lymphocytes affects AD symptoms [38]. Mutations in the filaggrin gene increase the incidence of various allergic diseases, including epidermal barrier defects and increased skin infections [39]. In addition, high levels of TSLP are expressed in AD, and T cells switch from Th1 to Th2 pattern [38, 39]. However, galectin-9 reduces mast cell degranulation and acute allergic skin reactions by promoting the differentiation of Th1 and Treg cells [40]. In this study, prebiotic treatment increased the population of Th1, Treg cells, and mRNA expression of galectin-9 in the MLNs isolated from DNCB-treated mice.

TLRs are expressed in intestinal epithelial cells and contribute to intestinal homeostasis [41], and especially, TLR-9 activation mediates innate and adaptive immunity [42]. TLR-9 has immunomodulatory effects via increased Th1 and Treg cells, resulting in alleviation of allergic diseases [27]. Using intestinal epithelial cell lines, we confirmed that prebiotic treatment increased the expression of TLR-9 and galectin-9 proteins.

Intestinal symbiotic microorganisms affect intestinal immunity via interaction between microbial antigens and pattern recognition receptors expressed by host cells [43]. Intestinal dysbiosis is a chronic inflammatory condition, similar to AD, and is associated with the onset of disease [44]. Thus, prebiotics modulate chronic inflammatory diseases such as AD by altering the composition of intestinal symbiotic microorganisms via immunomodulation. The intestinal microbial diversity is influenced by dietary behavior and has great influence on host physiology and immune function [45]. Westernized dietary habits associated with a reduction in dietary fiber intake result in reduced intestinal microbial diversity [46]. In the present study, we found that prebiotic treatment increased the intestinal microbial diversity. Consistent with our finding, previous studies have shown that bacterial species belonging to *Firmicutes* at the phylum level are increased in AD skin lesions [47], and the ratio of *Firmicutes* to *Bacteroidetes* is decreased in infants with food allergy [48]. In addition, *Clostridia* species appear to increase significantly at the class level [49]. Analysis of microorganisms belonging to *Firmicutes* revealed that *Lachnospiraceae* and *Ruminococcaceae* were dominant in the intestinal tract. AD-induced *Lachnospiraceae* and *Ruminococcaceae* decreased; however, following prebiotic treatment, the ratio of the two microorganisms was restored to normal levels.

It was reported that *Lachnospiraceae* was involved in the pathogenesis of allergy in infants, and *Ruminococcaceae* was low in infants with eczema-associated symptoms of IgE [34]. Butyrate is produced by fermentation of dietary fiber by intestinal microorganisms and contributes to intestinal homeostasis by increasing differentiation into Treg cells [50, 51]. In addition, butyrate-producing microorganisms regulate signal transduction to increase IgE production and increase the expression of IL-10, resulting in a reduction in antigen-specific allergic inflammatory responses in the intestine [52]. In this study, the butyrate-producing microorganisms belonging to *Ruminococcaceae* [53] were increased in the intestine of AD mouse treated with prebiotics. Additionally, we found that one of the butyrate-producing microorganisms, *Roseburia* spp. [54], and lactic acid bacteria were also increased after prebiotic treatment [55].

Previous studies have shown that reduced levels of *Clostridium clusters XIVa* and *IV* were associated with the development of AD [56,57]. Consistently, we found that AD induced *Ruminococcus* (genus) and *Ruminococcus lactis* (species) belonging to *Clostridia cluster XIVa* and *Anaerofilum* (genus) belonging to *clostridia cluster IV* were increased by prebiotic treatment.

In conclusion, prebiotic supplementation inhibits AD-like skin lesions in mice. Our results show that the inhibitory effect of prebiotics on AD is mediated via modulation of intestinal homeostasis as well as Th1/Th2 cytokine balance. These results suggest that prebiotics may have potential applications in the treatment of AD via attenuation of the allergic responses.

## Supplemental Materials



Supplementary data for this paper are available on-line only at http://jmb.or.kr.

## Figures and Tables

**Table 1:** RT-PCR primer sequences for the analysis of immunomodulatory genes.

Target gene	Primer		Reference
GAPDH	Forward	5’ CCACCCAGAAGACTGTGGAT 3’	15
	Reverse	5’ CACATTGGGGGTAGGAACAC 3’	
T-bet	Forward	5’ TCAACCAGCACCAGACAGAG 3’	16
	Reverse	5’ AAACATCCTGTAATGGCTTGTG 3’	
GATA-3	Forward	5’ CATTACCACCTATCCGCCCTATG 3’	16
	Reverse	5’ CACACACTCCCTGCCTTCTGT 3’	
RORγT	Forward	5’ TTCACCCCACCTCCACTG 3’	16
	Reverse	5’ TGCAAGGGATCACTTCAATTT 3’	
Foxp3	Forward	5’ CCCATCCCCAGGAGTCTTG 3’	17
	Reverse	5’ CCATGACTAGGGGCACTGTA 3’	
INF-γ	Forward	5’ TCAAGTGGCATAGATGTGGAAGAA 3’	17
	Reverse	5’ TGGCTCTGCAGGATTTTCATG 3’	
IL-4	Forward	5’ ACAGGAGAAGGGACGCCAT 3’	17
	Reverse	5’ GAAGCCCTACAGACGAGCTCA 3’	
IL-17	Forward	5’ TTCATCTGTGTCTCTGATGCT 3’	17
	Reverse	5’ TTGACCTTCACATTCTGGAG 3’	
TGF-β	Forward	5’ GAAGGCAGAGTTCAGGGTCTT 3’	17
	Reverse	5’ GGTTCCTGTCTTTGTGGTGAA 3’	
Galectin-9	Forward	5’ GAGAGGAAGACACACATGCCTTTC 3’	18
	Reverse	5’ GACCACAGCATTCTCATCAAAACG 3’	
Filaggrin	Forward	5’ CACTGAGCAAAGAAGAGCTGAA 3’	19
	Reverse	5’ CGATGTCTTGGTCATCTGGA 3’	
TSLP	Forward	5’ AGAGAAGCCCTCAATGACCAT 3’	19
	Reverse	5’ GGACTTCTGTGCCATTTCC 3’	

**Table 2:** qPCR primer sequences for the analysis of beneficial bacteria.

Target microorganism		Primer	Reference
Universal	Forward	5’ GTGSTGCAYGGYYGTCGTCA 3’	20
	Reverse	5’ ACGTCRTCCMCNCCTTCCTC 3’	
*Ruminococcus* spp.	Forward	5’ GGCGGCYTRCTGGGCTTT 3’	21
	Reverse	5’ CCAGGTGGATWACTTATTGTGTTAA 3’	
*Roseburia* spp.	Forward	5’ GCGGTRCGGCAAGTCTGA 3’	21
	Reverse	5’ CCTCCGACACTCTAGTMCGAC 3’	
*Faecalibacterium prausnitzii*	Forward	5’ GGAGGAAGAAGGTCTTCGG 3’	21
	Reverse	5’ AATTCCGCCTACCTCTGCACT 3’	
*Bifidobacterium* spp.	Forward	5’ TCGCGTCYGGTGTGAAAG 3’	22
	Reverse	5’ GGTGTTCTTCCCGATATCTACA 3’	
*Lactobacillus sakei*	Forward	5’ CCATGTGTAGCGGTGAAATG 3’	23
	Reverse	5’ ATCCTGTTTGCTACCCATGC 3’	
*Leuconostoc mesenteroides*	Forward	5’ TGATGCATAGCCGAGTTGAG 3’	23
	Reverse	5’ GAAAGCCTTCATCACACACG 3’	
*Leuconostoc citreum*	Forward	5’ GGAAACAGATGCTAATACCGAATA 3’	23
	Reverse	5’ TTTACCCCACCAACTAACTAATG 3’	
*Weissella cibaria*	Forward	5’ GGGAAACCTACCTCTTAGCA 3’	23
	Reverse	5’ GGACCATCTCTTAGTGATAGCA 3’	
*Weissella koreensis*	Forward	5’ GGGCTACACACGTGCTACAA 3’	23
	Reverse	5’ GATTCCGACTTCGTGTAGGC 3’	

**Table 3:** Effects of dietary prebiotic supplementation on diversity of fecal microbiota in mice.

Items	Treatments					

	C	N	P	T1	T2	T3
Genus	237 (2)	189 (12)^**^	187 (4)^**^	256 (23)^##^	250 (13)^##^	242 (9)^##^
Species	660 (20)	553 (10)^**^	554 (16)^**^	765 (51)^**##^	772 (22)^**##^	748 (73)^##^

C: Untreated control, N: AD control (DNCB-induced), P: Zyrtec-positive control (DNCB-induced + Zyrtec), T1: scGOS/lcFOS (DNCB-induced + scGOS/lcFOS), T2: Inulin (DNCB-induced + Inulin), T3: β-glucan (DNCB-induced + β-glucan); ^*^p < 0.05 versus control group; ^**^p < 0.01 versus control group; ^#^*p* < 0.05 versus negative control group; ^##^*p* < 0.01 versus negative control group. Data represent means ± SD of 4 replicates.

**Table 4:** Pyrosequencing analysis of fecal microbiota composition in mice fed with prebiotics.

Items	Treatments					

	C	N	P	T1	T2	T3

	mean % (SD)	mean % (SD)	mean % (SD)	mean % (SD)	mean % (SD)	mean % (SD)
***Firmicutes (p)***	81.38 (3.34)	87.82 (1.29)^*^	79.52 (2.22)^##^	63.59 (5.68)^**##^	76.12 (7.89)^#^	63.58 (6.21)^**##^
***Clostridia (c)***	65.67 (8.08)	83.05 (3.59)^**^	78.24 (0.83)^*#^	55.26 (7.32)^##^	74.33 (7.48)	56.45 (3.06)^##^
*Lachnospiraceae (F)*	42.76 (7.83)	70.47 (2.96)^**^	69.38 (2.08)	39.89 (3.06)^##^	47.37 (4.82)^##^	30.39 (6.62)^##^
*Eisenbergiella (G)*	7.89 (1.37)	7.53 (0.54)	7.98 (1.38)	8.91 (2.65)	14.14 (1.74)^**##^	5.45 (1.18)^*#^
*Ruminococcaceae (F)*	21.78 (4.98)	9.12 (0.59)^*^	8.93 (0.77)^**^	18.46 (3.91)^#^	20.54 (3.47)^##^	21.77 (3.72)^##^
*Ruminococcus (G)*	1.91 (0.67)	0.00 (0.00)^*^	0.00 (0.00)^*^	2.98 (0.66)^##^	3.02 (0.72)^##^	2.57 (1.30)^#^
***Bacilli (c)***	14.82 (8.22)	4.66 (4.80)	1.11 (1.73)^*^	4.26 (3.84)	5.02 (4.11)	2.94 (1.47)
*Lactobacillaceae (F)*	20.63 (7.96)	2.00 (1.52)^**^	0.32 (0.26)^*^	5.77 (3.35)^*^	4.32 (1.43)^*^	2.78 (1.42)^**^
*Lactobacillus (G)*	19.86 (7.69)	1.97 (1.49)^**^	0.31 (0.26)^*#^	5.53 (3.17)^*^	4.11 (1.37)^*^	2.66 (1.32)^*^
***Bacteroidetes (p)***	16.81 (3.77)	9.22 (1.79)^*^	16.41 (2.42)^##^	33.47 (5.26)^**##^	21.56 (7.96)^#^	31.89 (9.12)^*##^
***Bacteroidia (c)***	16.80 (3.78)	9.22 (1.79)^*^	16.40 (2.42)^##^	33.47 (5.27)^**##^	16.22 (4.89)^#^	31.88 (9.11)^*##^
*Prevotellaceae (F)*	0.71 (0.09)	0.52 (0.20)	0.85 (0.11)^#^	26.02 (6.74)^**##^	2.52 (2.00)	26.09 (8.54)^*##^
*Prevotella (G)*	0.67 (0.08)	0.15 (0.01)^**^	0.40 (0.16)^*#^	24.47 (6.26)^**##^	2.28 (1.76)	24.41 (8.23)^*#^
*S24-7_f(F)*	8.59 (1.89)	4.82 (1.59)^*^	6.97 (0.83)	7.08 (1.56)	7.64 (0.59)^#^	4.51 (1.59)^*^

C: Untreated control, N: AD control (DNCB-induced), P: Zyrtec-positive control (DNCB-induced + Zyrtec), T1: scGOS/lcFOS (DNCB-induced + scGOS/lcFOS), T2: Inulin (DNCB-induced + Inulin), T3: β-glucan (DNCB-induced + β-glucan); ^*^p < 0.05 versus control group; ^**^p < 0.01 versus control group; ^#^*p* < 0.05 versus negative control group; ^##^*p* < 0.01 versus negative control group. Data represent means ± SD of 4 replicates.

**Table 5:** Clostridia cluster analysis of fecal microbiota in mice fed with prebiotics.

Items		Treatments					

		C	N	P	T1	T2	T3

		mean % (SD)	mean % (SD)	mean % (SD)	mean % (SD)	mean % (SD)	mean % (SD)
**Clostridia cluster XIVa**							
Genus	*Ruminococcus*	0.969 (0.526)	0.000 (0.000)^*^	0.000 (0.000)^*^	2.985 (0.661)^**##^	3.021 (0.718)^**##^	2.570 (1.300)^#^
	*Roseburia*	0.569 (0.238)	0.507 (0.325)	0.172 (0.060)^*^	0.249 (0.108)	0.648 (0.251)	0.221 (0.093)^*^
Species	*Roseburia*	0.010 (0.008)	0.000 (0.000)	0.000 (0.000)	0.029 (0.032)	0.174 (0.172)	0.009 (0.011)
	*intestinalis*						
	*Lachnospira*	0.002 (0.003)	0.000 (0.000)	0.000 (0.000)	0.036 (0.033)	0.182 (0.206)	0.309 (0.217)
	*pectinoschiza*						
	*Ruminococcus*	0.005 (0.001)	0.000 (0.000)^**^	0.001 (0.002)^*^	0.033 (0.044)	0.018 (0.019)	0.006 (0.010)^*##^
	*lactaris*						
	*Coprococcus*	0.004 (0.003)	0.002 (0.003)	0.000 (0.000)	0.009 (0.003)^#^	0.006 (0.002)	0.014 (0.013)
	*comes*					
	*Clostridium*	0.008 (0.006)	0.002 (0.001)	0.007 (0.002)^##^	0.004 (0.002)	0.002 (0.003)	0.002 (0.002)
	*symbiosum*						
**Clostridia cluster IV**							
Genus	*Anaerofilum*	0.009 (0.004)	0.000 (0.000)^*^	0.003 (0.001)^*##^	0.393 (0.381)	0.029 (0.017)^#^	0.054 (0.071)

C: Untreated control, N: AD control (DNCB-induced), P: Zyrtec-positive control (DNCB-induced + Zyrtec), T1: scGOS/lcFOS (DNCB-induced + scGOS/lcFOS), T2: Inulin (DNCB-induced + Inulin), T3: β-glucan (DNCB-induced + β-glucan); ^*^p < 0.05 versus control group; ^**^p < 0.01 versus control group; ^#^*p* < 0.05 versus negative control group; ^##^*p* < 0.01 versus negative control group. Data represent means ± SD of 4 replicates.
