# Supplementary material for: Galectin-9 Induced by Dietary Prebiotics Regulates Immunomodulation to Reduce Atopic Dermatitis Symptoms in 1-Chloro- 2,4-Dinitrobenzene (DNCB)-Treated NC/Nga Mice
Source: J Microbiol Biotechnol. 2020 Jul 23;30(9):1343–54. doi: 10.4014/jmb.2005.05017 (PMC9745654; doi:10.4014/jmb.2005.05017)
Supplement: Supplementary file 1 [file JMB-30-9-1343-supple.pdf]

https://doi.org/10.4014/jmb.2005.05017

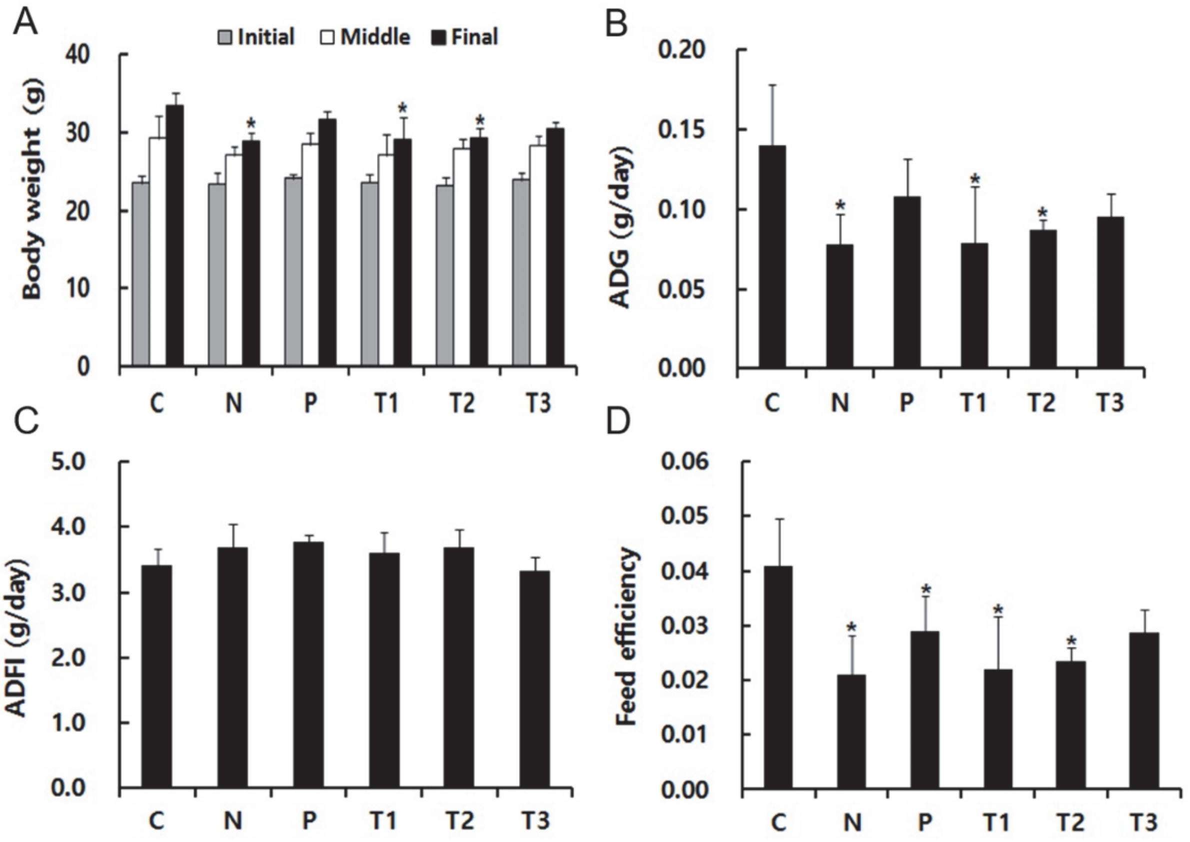

Supplementary Fig. 1. Effect of increasing percentage of dietary prebiotics on growth performance of mice. (A) Body weight; (B) average daily gain; (C) average daily feed intake; (D) Feed efficiency; C: Untreated Control; N: AD control (DNCB-induced); P: Zyrtec-positive control (DNCB-induced + Zyrtec); T1: scGOS/lcFOS (DNCB-induced + scGOS/lcFOS); T2: Inulin (DNCB-induced + Inulin); T3: β-glucan (DNCB-induced + β-glucan); *p < 0.05 versus control group; $^{\#}p$ < 0.05 versus negative control group. Data represent means ± SD of 4 replicates.
